# On Facial Hemiplegia and Paralysis of the Facial Nerve

**Published:** 1866-02

**Authors:** Wm. R. Saunders

**Affiliations:** M. D., F. R. C. P. Ed., Physician to the Royal Infirmary of Edinburgh, and Lecturer on Physiology and on Clinical Medicine


### ON FACIAL HEMIPLEGIA AND PARALYSIS OF THE FACIAL NERVE.

By WM. R. SAUNDERS, M.D., F.R.C.P.Ed., Physician to the Royal Infirmary of Edinburgh, and Lecturer on Physiology and on Clinical Medicine.

*Centric and facial peripheral paralysis.—Dr. Todd's views.—Objections critical and clinical.*

Since the discoveries of Bell, it has been well known that paralysis of the fascial muscles may be produced by one or two causes. Either the palsy is due to a cerebral lesion (cerebral or centric fascial hemiplegia,) in which case it is usually accompanied by hemiplegia of the limbs on the same side; or, occurring independently of cerebral disease, it is owing simply to lesion of the trunk or branches of the portio dura at some part of its course (peripheral facial hemiplegia, paralysis of the facial nerve, Bell's paralysis). These two kinds of facial palsy present many points of resemblance and of contrast, which are of considerable interest and importance. My present purpose is —1st, to direct attention to certain views in regard to them, which, although held upon high authority, seem to me to be erroneous; and 2d, to endeavor to explain some difficulties which have not yet received a satisfactory solution.

I. The opinions I refer to, which I believe to be mistaken, assert that in facial hemiplegia due to a cerebral lesion, the nerve which is usually paralysed is not the facial of the seventh pair, as many believe, but, on the contrary, is the motor portion of the fifth pair. This doctrine was long ago maintained by Mr. John Shaw, brother in-law of Sir Charles Bell; but it has been principally brought into notice and powerfully enforced in recent times by the late distinguished physiologist and physician, Dr. Todd in his Clinical Lectures on Nervous Diseases. Supported by his eminent authority, this peculiar view has been widely accepted in England, and has been latterly put forward as an established truth in our best text-books on the Practice of Medicine—*e.g.*, those of Dr. Watson and Dr. Aitken.* The subject is of sufficient importance to deserve investigation; and after careful examination of it, I am unable to consent to Dr. Todd's opinion. On the contrary, it appears to me that his peculiar views can be disproved, and the source of their error discovered, by an examination of Dr. Todd's own statements, and by investigating the facts supplied by clinical observation.

* Also, Tanner, "Practice of Medicine," Fifth Edition, published since this paper was written.

*Examination of Dr. Todd's statements.*—The symptoms of fascial palsy due to lesion of the seventh pair are too well known to need recapitulation. It will be sufficient to quote Dr. Todd's description of the phenomena of facial hemiplegia from cerebral disease, as distinguished from these of peripheral paralysis of the portio dura, in order fully to represent the principal facts, as well as his peculiar views on the subject. In his forty-first lecture, on Hemiplegia, p. 709 (second edition, by Beale), he says:—

"It is very necessary that you should be well impressed with the characters of facial palsy which accompanies hemiplegia, and that you should be able readily to distinguish it from that which arises from affection of the portio dura. Place the patient well opposite to you, and observe the condition of the face as it is perfectly quiescent. You will observe that *the paralysed cheek hangs*, and that the *angle of the mouth* on that side is *lower* than its fellow; the *cheek* is more or less *loose* and *flaccid*, in proportion as the paralysis is more or less perfect. If now you ask him to smile or speak, *the want of equilibrium of the face* becomes very apparent. The healthy muscles, being relieved of the antagonism of the paralysed ones, contract to a much greater extent than is natural; while the *palsied cheek remains quiescent*, or allows itself to be *drawn* slightly towards the mesial line.

He then alludes to the distortion of the features as observed by the patient or his friends and continues:—

"But, with all this, the patient can *shut both eyes well and open them*, and he can *move the cheek* so far as can be effected by the zygomatic muscles, and he makes a very fair *attempt* at pursing up the mouth for whistling; the last act, however, being *rather impaired* by the intimate connection of the buccinator with the orbicular muscle of the mouth. The *muscles of mastication* on the paralysed side act with *less power*, although *it seldom happens* in hemiplegia that their power is completely *destroyed. Doubtless much of this action is reflex*, and this explains the fact that *while the buccinator is very much paralysed and even wasted, the masseter and other masticatory muscles retain a considerable amount of power.*"

The conclusions which Dr. Todd arrives at from an examination of the symptoms described are thus given:—

"Your anatomical knowledge will explain all this to you: *the fifth nerve is more or less involved in, or influenced by, the paralysing lesion.*" And further on: "*The facial nerve, or portio dura, is not generally touched by the paralysing lesion in*

*hemiplegia,*" although in a few cases "the muscles were weakened, as if the nerve participated slightly in the shock------In a physiological point of view," he adds afterwards "it is a very interesting inquiry how it happens that the fifth and ninth nerves are so frequently—nay, almost universally—paralysed in hemiplegia, while the portio dura escapes."—p. 711.

Notwithstanding the positive and decided terms in which Dr. Todd announces these conclusions, it appears to me that no physiologist or physician can read his description, just quoted, of the symptoms of facial hemiplegia, without perceiving plainly that, instead of supporting, they are opposed to the interpretation he puts upon them. For—

1st. Amongst the symptoms described, those of paralysis of the muscles supplied by the portio dura of the seventh pair are undoubtedly present.

It is anatomically impossible that the *hanging of the paralysed cheek*, *depression of the angle of the mouth*, *distortion of the features*, *exaggerated when the patient speaks or smiles*, could be produced by paralysis of the muscles of mastication, which are alone supplied by the fifth pair. These are symptoms of inaction of the facial muscles of expression, and necessarily imply paralysis of the nerve which supplies them with motion—viz., the seventh pair, and no other. On this point there is no room for doubt. It will be observed that Dr. Todd makes the qualifying statement, that the patient can move the cheek so far as can be effected by the zygomatic muscles, and can make an attempt at pursing up the mouth for whistling. But it is well known that in cerebral hemiplegia, the paralysis is seldom so complete as to prevent all movement in the parts affected; and further, we cannot conceive the action of the zygomatics to have been very apparent when the *cheek hung locse*, and remained *quiescent when the patient smiled.* The attempt at pursing the lips for whistling is also noted as "impaired,"—the impairment being ascribed, it should be remarked, to the connexion of the *buccinator* with the orbicular muscles of the mouth. The nature and extent of the paralysis of the buccinator and of the orbicularis palpebarum will, however, fall to be fully examined presently, and it will then appear what is really implied in this explanation of the impairment in whistling.

2nd. While the symptoms of paralysis of the motor seventh are, contrary to Dr. Todd's statement, undoubtedly present, those indicating paralysis of the fifth are not satisfactorily demonstrated.

It must be first noted that Dr. Todd looks upon the buccinator as a muscle of mastication supplied with motor power by the fifth pair. The objections to this will be stated afterwards. Meanwhile we observe that the evidence adduced of motor paralysis of the fifth nerve consists in this, that the muscles of mastication (including the buccinator) "act with less power, although it seldom happens that the power is completely destroyed." For, "while the buccinator muscle is very much paralysed, and even wasted, the masseter and other masticatory muscles retain a considerable amount of power." It is not easy to understand, from Dr. Todd's point of view, how the marked distortion of the face which he describes could, if possible at all, be produced by so small an amount of paralysis of the muscles of mastication. Further, while he ascribes the distortion of the features to paralysis of the fifth, which, it can be shown that paralysis could not produce, he says nothing of falling or obliquity of the lower jaw, which that paralysis would certainly occasion. Finally, to account for the minor degree of masticatory paralysis (to which nevertheless he ascribes all the facial symptoms), and to explain the exemption of the masseter, &c., from the atrophy which effects the buccinator, he resorts to the extraordinary assumption that "much of the action" of the masticatory muscles (*i.e.*, of all except the buccinator) "is reflex"—a strange hypothesis, which the misunderstanding of the nervous supply of the buccinator, and the necessity of making his theory consistent, could alone have originated.

It is proper to add that in one case, that of Coulson, cited by Dr. Todd (p. 710), motor paralysis of the fifth nerve was observed, and was proved by wasting of the temporal and masseter muscles; but that this case, otherwise complex, was of an exceptional kind is sufficiently evinced by Dr. Todd's own statement already noticed, that in ordinary cases of hemiplegia, even when the buccinator wastes, there is no atrophy of the masseter and temporals. The exception, by its marked contrast, serves to prove the rule, which affirms the rarity of affection of the motor fifth in ordinary hemiplegia.

*Clinical observation.*—The evidence of clinical observation is no less opposed to Dr. Todd's views than the anatomical and physiological considerations which his own statements have suggested. I have carefully investigated the facial phenomena in cases both of peripheral paralysis of the portio dura and of facial hemiplegia due to cerebral disease, which have occurred in the wards of the Edinburgh Royal Infirmary under my charge. In all instances hitherto, I have found that in ordinary facial palsy of cerebral origin, the muscles of expression, including the buccinator, were more or less affected; while the action of the masseters, temporals, and pterygoids was unimpaired. The usual facial distortion was exhibited in a greater or less degree, according to the amount of the paralysis, although it rarely approached the completeness usually seen in peripheral paralysis from lesion of the nerve-trunk. The movements of smiling and of labial articulation were impeded on the paralysed side; mastication was rendered difficult so far as the action of the buccinator was concerned, but the proper masticatory movements of the jaw were vigorous and equal on the two sides. I could observe no defect or want of force in the muscles of the jaw on the paralysed side so that the weakness of the buccinator presented a marked contrast to their condition. There was no hanging down, and no lateral displacement or obliquity of the inferior maxilla, either at rest or during mastication. The mesial line between the lower incisors correspond accurately to that in the upper jaw; while on the contrary, the middle line of the lips was drawn to the sound side. As usually occurs in cerebral hemiplegia, the orbicularis palpebrarum muscle was little affected; the eyelids on the paralysed side could not be closed so firmly as on the opposite side, but the voluntary closure could take place, and the act of winking was unimpaired. Of this speciality of the orbicularis palpebrarum muscle I shall afterwards attempt an explanation. For the present, it is sufficient to observe that, with this exception, the phenomena of cerebral hemiplegia of the face are entirely similar to those of facial paralysis, produced by lesion of the seventh nerve itself, and, as in the latter, are confined to the muscles of expression, including the buccinator, and do not involve the masticatory muscles (masseter, temporal, pterygoid) supplied by the motor fifth. After a candid investigation of such cases, it appears to me as if no reasonable doubt can remain that in cerebral hemiplegia, when the face is affected, it is the motor branch of the seventh pair, and not (as Dr. Todd would have us believe) the fifth nerve, which is paralysed of motion. It is unnecessary and would be tedious to record individual cases; I shall content myself by referring to one of hemiplegia depending on cancer of the brain, in which the above facts were carefully noted, and the particulars of which are briefly referred to in the report of the Proceedings of the Medico-Chirurgical Society of Edinburgh, before which I exhibited the morbid specimen at their meeting on the 1st March, 1865; commenting at the same time upon this and other similar cases in their bearings on Dr. Todd's views. I beg also to refer, in confirmation of the clinical evidence, to the cases of diseases of the nervous system recently published by Dr. Hughlings Jackson, in the first volume of the Clinical Lectures and Reports of the London Hospital, 1864. I am much indebted to Dr. Jackson for lately directing my attention to his cases and remarks on the subject.

I shall only add, that, while Dr. Todd's views have been readily accepted in England, I am not aware of any Continental author who has adopted or who has even appeared to be acquainted with them. The current doctrine abroad is the one which the preceding considerations lead me still to look upon as correct—that in cerebral hemiplegia, as in peripheral face-palsy, it is the motor seventh nerve which is affected. At the same time it should be observed that foreign authors have recognized, but have not explained, the differences in the phenomena of the centric and peripheral forms of facial paralysis which formed the foundation of Dr. Todd's peculiar views.

—

*Examination of difficulties in regard to buccinator and orbicularis palpebrarum muscles.—Loss of galvanic contractility in muscles in peripheral hemiplegia.—Other points requiring investigation.*

II. It now remains to inquire what were the points of difficulty in the phenomena of facial hemiplegia which induced so distinguished an observer as Dr. Todd to adopt his peculiar views, and by which his opinions are still by some conceived to receive support. It is apparent, from what has preceded, that these difficulties are chiefly two: one of them science has long since solved, while the other still remains to be explained. The first anomaly is the affection of the *buccinator* muscle, which is paralysed both in palsy of the portio dura and in cerebral hemiplegia of the face; the second in the partial exemption of the *orbicularis palpebrarum* muscle, which is affected in peripheral paralysis of the portio dura, but escapes injury in the facial palsy of cerebral origin.

The *buccinator* muscle stands in the peculiar anatomical relation that it is supplied both by the fifth and by the seventh nerves; and, till recently, anatomists regarded the buccal branch of the fifth as either the only or the chief motor nerve of this muscle. Dr. Todd held the view that the buccinator received motor branches from both nerves, and he accounted for the double supply on the hypothesis that the facial was the motor nerve of the buccinator as a muscle of expression, while the fifth communicated the motor influence for mastication only. This hypothesis led to serious confusion and difficulty, which

Dr. Todd felt, but from which he could not extricate himself. He confesses himself unable to understand why the buccinator muscle should be completely paralysed, even of its masticatory function, in lesion of the portio dura, which he held to be its motor nerve for expression only; and he was at no less loss to know why it was paralysed, as a muscle of expression, in what he assumed to be paralysis of the fifth pair, in cerebral hemiplegia. Of these, which he calls "curious facts," he hints at a possible explanation, in accordance with Marshall Hall's views, that the fifth nerve might possess a cerebral and the seventh a spinal character; but he at once rejects the solution as "inadequate to explain the complete palsy of the buccinator muscle when the fifth is the only nerve affected, as in common hemiplegia" (p. 649), and leaves the "facts" unexplained, and apparently inexplicable.

It is now more than twenty years since the error and difficulty in regard to the buccinator muscle was clearly corrected and explained, on the basis both of anatomical and physiological science, and of clinical observation, by M. Longet in his celebrated work "Anatomie et Physiologie du Systeme Nerveux" (Paris, 1842, ii., p. 187, and p. 408 *et seq*).* The experiments of succeeding physiologists, and the observations of physicians, have confirmed M. Longet's statements; and this point of physiological anatomy is so far settled that it is now usually accurately stated in our best ordinary text-books of physiology. The correct account is that the seventh pair is the only motor nerve of the buccinator muscle for all its actions, whether of expression or of mastication; and that the fifth pair supplies it, not with motor, but with sensory fibres. It is equally strange that Dr. Todd should have been unacquainted with Longet's observations, or that he should have entirely omitted to consider them. But it is, nevertheless, the case that not only Dr. Todd, but the principal anatomical and physiological authors and lecturers, with few exceptions, continued up to the last few years, and many still continue, to teach the erroneous doctrine that the buccinator muscle is supplied with motion by the fifth pair, and not by the seventh.† Amongst the few who taught correctly, I must not omit to mention my friend Dr. Struthers, now Professor of Anatomy in Aberdeen, who informs me that, at an early period, he became convinced, by the independent observation of clinical cases of paralysis of the portio dura, that

* Also previously by Mayo, 1823; and Volkmann, in Muller's Archiv, 1840.

† Physicians also commonly make this mistake; even the learned and accurate Hasse, in Virchow's Handbuch der Path u. Ther., Bd. iv., 343.

the motor supply of the buccinator was derived exclusively from that nerve. He has, accordingly, always so taught in his lectures, and had made a collection of clinical cases illustrative of the point, the notes of which he has obligingly forwarded to me. Not to dwell unnecessarily on this point, however, let it be understood plainly, that *the buccinator muscle is interrupted in all its functions, whether of expression or of mastication, whenever the portio dura is paralysed; it is unaffected, and all its actions are preserved, in motor paralysis of the fifth pair.* It will be perceived that this completely explains the difficulties in regard to the buccinator which Dr. Todd considered insuperable; but, at the same time, it entirely subverts his notion that paralysis of the buccinator in cerebral hemiplegia is due to lesion of the fifth nerve, and it destroys one of his main arguments for maintaining that the fifth nerve is commonly affected in that form of paralysis.

In regard to the orbicularis palpebrarum muscle, another source of difficulty appeared. In facial palsy from lesion of the portio dura, the orbicularis palpebrarum is almost invariably paralysed; the voluntary closure of the eyelids, the usual movements of winking and the shutting of the eye when the conjunctiva is irritated, all are more or less interrupted or prevented. On the contrary, in the great majority of cases of cerebral hemiplegia of the face, the orbicularis palpebrarum is not materially affected; the act of winking and of voluntary closure of the eyes continues on the paralysed as on the sound side, with the small exception that the voluntary closure is usually weaker on the palsied side. These symptoms, indeed, form one of the best diagnostic marks between centric and peripheral paralysis of the face: the hanging cheek, with wide-open, staring, unwinking eye, denotes lesion of the portio dura; the flaccid face, with the natural position and motions of the eyelids, is the sign of cerebral lesion, and indicates a more serious disease. This valuable distinction is said to have been first noted by Recamier,* its patho gnomonic value is insisted on by Dr. Todd (p. 644), and it is well known to every clinical observer. There can be but little doubt that the usual immunity of this orbicular

* Duchenne; Electrisation Localisee, 2e Edit., 1861, p. 365, note; where it is also stated that M. Duplay, in *l' Union Medicale* of Aug. 28th, 1854. "De la paralysie faciale, produite par une hemorrhagie cerebrale chez les vieillards," has reported several cases of hemiplegia, in which a cerebral cause was demonstrated on dissection, and in which nevertheless the orbicular muscle of the eyelids was paralysed. I regret that I have no means of access to this paper, as its study would probably be peculiarly interesting and instructive in relation to the present inquiry.

muscle (which is supplied by the seventh pair) in cerebral hemiplegia affecting the face, while it is so conspicuously paralysed in the peripheral paralysis of the portio dura, was one of the main reasons which led Dr. Todd to assume that in the former case it was not the seventh but the fifth nerve that was at fault. And we have heard the same fact alleged recently in support of Dr. Todd's opinions, or at least as still unaccounted for on the supposition that the seventh is the nerve affected in cerebral hemiplegia. It is important therefore to search for a solution of the difficulty.

In examining this question, it is necessary to remember that one reason why the action of the orbicularis palpebrarum is not arrested in cerebral hemiplegia is, because ordinary winking is a reflex and not a voluntary act. When the motor influence of the brain is withdrawn from a part, the rule is, that the voluntary movements cease, but the reflex action persist. Winking continues therefore in cerebral hemiplegia of the face after the mobility of features has ceased, for the same reason that respiratory movements of the intercostals and diaphragm are preserved in the cerebral hemiplegia, which deprives the limbs of voluntary power. On the other hand, both voluntary and involuntary closing of the eyelids is prevented, and the orbicularis palpebrarum and other muscles are completely paralysed in lesion of the portio dura, because the efferent conduction of all motor influence, reflex and voluntary alike, is then interrupted. In the same way, spinal hemiplegia is distinguished from cerebral by the muscles of the thorax being paralysed in conjunction with those of the limbs, because in the spinal disease the tracts for reflex action are injured simultaneously with the conductors of volition.

This explanation which accounts for part of the phenomena, may be extended, however, beyond the simple reflex actions, as follows:—

The facial motor or portio dura of the seventh pair, considered as a musculo-motor nerve, contains within its common trunk fibres serving different purposes, and provided therefore with different capacities of action. These fibres may, for our present inquiry, be divided into—1st, voluntary motor fibres, by which the voluntary movements of the features are performed, and by which especially labial and buccal speech and mastication are accomplished; 2nd, emotional fibres, by which the features express the passions more or less involuntary; 3rd, reflex motor fibres, which are involuntary for the act of winking and for the movements of the nostrils in respiration.

According to the principles of physiology, these different sets of fibres derive their peculiar functions, not from any special properties of the nervous fibres themselves, but solely from the nature of their origin or central connexions in the brain or medulla oblongata. Thus, although anatomy cannot yet trace individual fibres to their primary source there is good reason to believe that the voluntary motor fibres originate from a cerebral centre of conscious volition: those for expression must be connected with the cerebral organs of the emotions; while those for reflex action arise, more or less independently of the cerebral hemispheres, from the medulla oblongata, in connexion (*inter alia*) with the sensory branches of the fifth pair, with which they are associated in the act of winking.

If these propositions are admitted, it is clear that, in lesion of the nerve-trunk in which all the fibres indiscriminately are equally liable to be affected, not only voluntary, but emotional and reflex motions will be suspended. But when the cause of the paralysis is cerebral, the origin or course of certain sets of fibres may alone be involved by the lesion, while that of other sets, being in a different part of the encephalon, may entirely escape direct injury; and the symptoms will vary with the special seat of the central disease. The voluntary and emotional actions, either or both, which have their origin and in the cerebrum, will usually suffer; while the fibres for reflex action which have their source in the medulla oblongata, may be expected to retain their power. The play of the features will be lost, and buccal and labial speech and mastication impaired; but the natural position of semi-closure, and the involuntary winking of the eyelids will be preserved. In short, as there are three sets of fibres to be taken into account, so there are three species of paralysis of the facial nerve: voluntary motor paralysis, emotional parlaysis, reflex paralysis which may or may not co-exist.

In tracing out these views, which to some may appear too theoretical, I was not aware that any cases had been observed of centric facial hemiplegia, where the voluntary and emotional actions had been separately and independently paralysed. On referring to Romberg's work, however, I found two very remarkable cases quoted of this description. In the one case reported by Magnus,* the voluntary movements of the face were entirely lost, while the facial muscles still acted powerfully under the influence of emotion. The other case by Stroymeyer (of which I have been unable to see the original in Casper's

* Muller's Archiv for 1837, pp. 258-266, and p. 567.

"Wöchenschrift für die Gesammte Heilkunde," (1837, p. 33) is the converse of Magnus's: there was paralysis of expression and of respiratory movements on the right side of the face, with the usual facial distortion, increased by emotions or talking: but the voluntary power remained, for the patient was as able to "control the muscles on this side as those on the left. She could move the angle of the mouth, dilate her nostrils, wrinkle her forehead, and contract her eyebrows at will," &c. Magnus explains this case by supposing that the emotional impulse being the stronger, could overcome an obstacle to nervous conduction which resisted the effort of volition. But Stroymeyer's case is opposed to such a supposition. On the theory of separate nerve-centres, such as I have indicated above, both cases would be readily explained.

I should not wish to urge these theoretical views too far, nor to attach more value to them than this, that they afford an explanation in accordance with the present state of nervous physiology and pathology, and still more that they suggest further inquiry into facts, especially dissections of cerebral hemiplegia, which may, in confirming or confuting them, extend our knowledge.

Whether explicable on the principles just referred to or not, it is necessary to attend to the fact, that while the facial palsy due to lesion of the nerve is usually complete, that caused by crebral disease is nearly alway incomplete, both in the number of muscles affected and in the degree of their paralysis; the affected muscles are weakened and disabled, but have not wholly lost their power. The same partial and incomplete character is observed in cerebral hemiplegia of the limbs, the arm being usually more affected than the leg, &c. In the face it is usually the lips and cheek that are most affected, causing difficulty of articulation and mastication, and distortion on smiling; although careful observation will show at the same time diminished power in closure of the eyelids, wrinkling of the nose, &c. The partial and incomplete nature of the paralysis is further shown by the stimulus of galvanism: the galvanic contractility of muscles is greatly impaired, and is soon entirely lost, in lesion of the portio dura, while it is retained, or is only slightly enfeebled, in crerbral hemiplegia. The same difference of susceptibility to galvanic stimulus is observed in limbs, according as the brain or a nerve-lesion is the cause of the paralysis. Such facts are not inconsistent with the theory of different centric origin of several sets of fibres bound up together in the same nerve-trunk, and they afford no ground for supposing that it is the fifth nerve, and not the seventh, which is paralysed in the ordinary cases of cerebral hemiplegia involving the face. They merely indicate that a centric lesion may involve a part only of the origins of the nerves affected, and may damage only, and not destroy even these.

Having thus discussed, so far as my present opportunities admit, the points of difficulty which had occurred to Dr. Todd and other observers, I have only further to remark, that not only does it appear certain to me that in facial hemiplegia it is the seventh, and not the fifth nerve, which is affected, but I have hitherto failed to observe any clinical evidence that the fifth pair is at all implicated in ordinary cases of facial hemiplegia.* Complicated cases occur in which the fifth and the other cerebral nerves, one or more, are involved in an intra-cranial disease; but in the ordinary cases of cerebral hemiplegia of the face, to which Dr. Todd's statement alone refers, the rule is the reverse of what he asserts: the facial is paralysed, the fifth nerve is exempt.

There are certain other points of interest which I shall not enter upon, but to which I should wish to call the attention of physiologists and of clinical observers. These are,—

1st. Why the contractility of muscles under the galvanic stimulus is speedily lost in peripheral paralysis of the portio dura, while it is retained in cerebral hemiplegia of the face? What bearing has this fact on the doctrine of the *vis insita?*

2nd. Clinical evidence of reflex paralysis of the seventh pair from impairment or lesion of the sensory portion of the fifth pair.

3rd. Why, and under what conditions, is facial paralysis succeeded by contracture or tonic spasm of the muscles previously paralysis?† I had recently under my care a patient affected with peripheral paralysis of the facial nerve, in whom the affected muscles had completely lost their contractility under the stimulus of galvanism, and in whom nevertheless, the previously palsied muscles are now the seat of contracture, as much over-shortened as they were previously over-lengthened. There has been, at the same time, some return of voluntary power in them. A casual observer seeing him for the first time, would think that the natural side of his face (the left) was paralysed, and that the opposite one exhibited the usual dragging of the features to its own side. But on voluntary or emotional action,

* Romberg, however, describes motor and sensory paralysis of the fifth pair as frequent in recent cerebral hæmorrhage. I know no evidence of this.—Sydenham Society Transactions, vol. ii., p. 289; and Muller's Archiv, 1838.

† Spasmodic tic of Marshall Hall, who first described it.

the motor power is found to be natural on the elongated left side of his face, and deficient on the shortened right one. Though the right eyelids are closer together than the left ones, he cannot by any effort close them completely, while they readily shut on the opposite side. What is the meaning of this contracture? Is it a return of muscular power in excess, or a kind of *rigor mortis?* It could only be the latter if it preceded wasting.

4th. The influence of the facial on vascularity (vaso-motion) and secretion. I have not in my cases seen any good examples of this.

5th. The influence on the palate, of which I have seen some marked examples. (See *Edinburgh Medical Journal* for August and September.

These, and several other points which this subject presents, have not been sufficiently investigated, and both scientifically and practically they are interesting and important.—*London Lancet.*

